# A graphical model approach to automated classification of protein subcellular location patterns in multi-cell images

**DOI:** 10.1186/1471-2105-7-90

**Published:** 2006-02-23

**Authors:** Shann-Ching Chen, Robert F Murphy

**Affiliations:** 1 Department of Biomedical Engineering and Center for Bioimage Informatics, Carnegie Mellon University, Pittsburgh, PA 15213, USA; 2 Department of Biological Sciences and Center for Automated Learning and Discovery, Carnegie Mellon University, Pittsburgh, PA 15213, USA

## Abstract

**Background:**

Knowledge of the subcellular location of a protein is critical to understanding how that protein works in a cell. This location is frequently determined by the interpretation of fluorescence microscope images. In recent years, automated systems have been developed for consistent and objective interpretation of such images so that the protein pattern in a single cell can be assigned to a known location category. While these systems perform with nearly perfect accuracy for single cell images of all major subcellular structures, their ability to distinguish subpatterns of an organelle (such as two Golgi proteins) is not perfect. Our goal in the work described here was to improve the ability of an automated system to decide which of two similar patterns is present in a field of cells by considering more than one cell at a time. Since cells displaying the same location pattern are often clustered together, considering multiple cells may be expected to improve discrimination between similar patterns.

**Results:**

We describe how to take advantage of information on experimental conditions to construct a graphical representation for multiple cells in a field. Assuming that a field is composed of a small number of classes, the classification accuracy can be improved by allowing the computed probability of each pattern for each cell to be influenced by the probabilities of its neighboring cells in the model. We describe a novel way to allow this influence to occur, in which we adjust the prior probabilities of each class to reflect the patterns that are present. When this graphical model approach is used on synthetic multi-cell images in which the true class of each cell is known, we observe that the ability to distinguish similar classes is improved without suffering any degradation in ability to distinguish dissimilar classes. The computational complexity of the method is sufficiently low that improved assignments of classes can be obtained for fields of twelve cells in under 0.04 second on a 1600 megahertz processor.

**Conclusion:**

We demonstrate that graphical models can be used to improve the accuracy of classification of subcellular patterns in multi-cell fluorescence microscope images. We also describe a novel algorithm for inferring classes from a graphical model. The performance and speed suggest that the method will be particularly valuable for analysis of images from high-throughput microscopy. We also anticipate that it will be useful for analyzing the mixtures of cell types typically present in images of tissues. Lastly, we anticipate that the method can be generalized to other problems.

## Background

The location (or locations) of a protein within cells is an important attribute that can be largely independent of its structure, enzymatic activity, or level of expression. Systematic and comprehensive analysis of subcellular location is therefore needed as part of systems biology efforts to understand the behavior of all expressed proteins. Work in this area can be divided into experimental *determination *and computational *prediction*. Of course, the accuracy and utility of prediction methods is dependent on the accuracy, coverage and resolution of determination methods. This is because experimentally determined locations are the starting point for the machine learning methods at the heart of prediction systems [1-3]. Subcellular location is most frequently determined by visual interpretation of fluorescence microscope images, but such interpretations can be highly variable from observer to observer. We have therefore developed automated systems to recognize major subcellular patterns [4-6] and to learn new patterns directly from fluorescence microscope images [7,8]. These systems utilize high resolution images and have been shown to be able to distinguish similar patterns better than visual examination [9].

### Automated interpretation of subcellular patterns in micrographs

**Figure 1:**
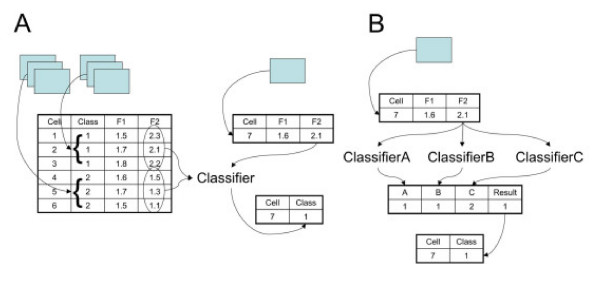
**Illustration of classification approaches to single cells**. A) Basic approach to feature-based classification of single cell images. B) Majority-voting classifier.

The automated location determination systems consist of machine classifiers (such as neural networks or support vector machines) and sets of informative numerical features (which we term SLFs for Subcellular Location Features) to describe protein distributions in the cell. This process is illustrated in Figure 1a. The starting point is the collection of many images of two (or more) different protein patterns. Regions containing single cells are identified either automatically or manually, and background fluorescence is subtracted. A number of different types of SLF are then calculated for each cell, including morphological features that describe the number, distribution, size and shape of fluorescent objects in the cell and texture features that describe the pixel-to-pixel variation in intensity. A feature matrix is then created in which each row shows the values of each feature for a given cell along with the type (or "class") of the protein that was labeled in that cell. This matrix is used to train a classifier so that it can learn a mapping between the SLF and the classes. For each new (test) cell, the process of segmentation, background subtraction, and feature calculation is repeated, and the feature vector is supplied to the classifier to assign the cell to one of the known classes.

Using large collections of HeLa cell images containing ten distinct subcellular patterns, our systems have achieved classification accuracies as high as 92% and 98% for 2D and 3D single cell images, respectively [10,11]. The patterns of dissimilar classes can be distinguished quite well; however, there is still room to improve the classification accuracy for similar classes (such as endosomal and lysosomal proteins and different Golgi proteins).

In order to improve the classification accuracy, one strategy is to incorporate additional or improved features and another is to combine more than one classifier using voting methods. The performance improvements we have obtained for 2D HeLa images, from 83% using a library of 84 features and a neural network classifier [6] to 92% using a library of 180 features and a majority-voting ensemble [10], resulted from implementing both of these strategies. A majority-voting ensemble combines the results from many different classifiers into a single decision, as illustrated in Figure 1b.

These improvements were obtained while considering the classification of patterns in single cells. An additional strategy is to utilize information from more than one cell from the same sample. For example, when sets of HeLa cells from the same slide were individually classified and allowed to vote for a single classification for the entire set, overall accuracy improved from 83% to 98% [6]. The penalty for this improvement is that we give up the ability to identify more than one pattern in a given set. A possible improvement on this approach is therefore to first estimate the number of classes that are present from the frequencies of the classes (by ruling out classes that have a low frequency), and then assign each cell to one of the remaining classes. (If we rule out all but one class, this approach reduces to the previous one.) So that we can decide which classes to rule out, we assume that the "true" classes are present in roughly equal proportions. In this paper, we first evaluate this simple strategy. We then describe more sophisticated approaches that construct a graphical model representing pattern information for more than one cell in a field so that improved classification accuracy can be achieved while retaining the ability to classify each cell individually (and without the assumption that classes are present in equal frequencies).

### Graphical models

Graphical models have been extensively applied to problems in the computer vision field, such as image segmentation and object recognition, where the pixels in an image can be segmented or classified into two (foreground and background) or more classes [12]. Many classification problems where the labels of related objects must be consistent with each other, such as hypertext classification [13] and identification of protein functions in the protein-protein interaction network [14], can also utilize graph-based methods. To our knowledge, graphical models have not previously been applied to the recognition of subcellular patterns in multi-cell images. Large numbers of such images are increasingly being acquired both in projects aimed at determining the subcellular location of all proteins [8,15-17] and in drug screening by high-throughput microscopy [18]. Part of the motivation behind the work we describe here is the need to classify fields of cultured cells that may be expressing different tagged proteins (such fields arise when a population of cells is randomly tagged). An additional motivation is the desire to classify individual cell patterns in tissues that may consist of more than one cell type.

The problem to be solved using a graphical model is to infer the posterior probability of each class for each node (cell) using information about the likely classes of other nodes (cells). For some graphical models, an exact solution can be found using the belief propagation (BP) algorithm [19]. However, BP can only calculate the posterior probability correctly on trees or forests, that is, on graphs where there is at most one path between any two nodes. If there are loops in the graph, the junction tree algorithm [20] can be used to convert a loopy graph into a tree by clustering nodes together. Exact inference can then be achieved by applying BP on the converted tree, but the running time is exponential in the size of the largest cluster in the converted graph. We therefore need approximate inference methods for cases where the size of the largest cluster is large. A commonly used approximate method is loopy belief propagation (LBP), which iteratively applies belief propagation updates on a graph with loops. LBP often gives good approximate inference when it converges [21], and often runs very quickly, but can fail to converge on some graphs. Other approximate inference algorithms, such as variational methods [22] and Monte Carlo methods [23], are also widely used. Running times for these approximate inference methods can be prohibitive for large graphs.

A graphical model consists of an algorithm for constructing the graph itself and an algorithm for making inferences given the graph. In this paper we describe how to construct graphs for the problem of subcellular location classification, and also present a novel algorithm, which we term prior updating, that permits inferences to be made for the (often large) resulting graphs.

## Results

*Problem Statement: *At the outset, we formalize our problem by describing our assumptions about the process used to create cell images. We assume that the process of creating a slide (or a well, plate or chamber) for imaging starts by creating a mixture of any number of cells from each of many possible classes. We further assume that cells are randomly distributed over the slide at some time *t*_*plate *_before imaging, that the cells divide with an average generation time of *t*_*g*_, and that the class of a cell is stably inherited by its daughters (the latter assumption can be relaxed slightly to allow for mutation without substantially changing our treatment). Lastly, we assume that we have accurate methods for segmenting multi-cell images into regions containing single cells, and classification methods that provide a likelihood for each possible class for each segmented cell. The task is: Given an image of a field containing a number of cells meeting the assumptions above, assign a class to each cell as accurately as possible.

### Equal-sized class model

As discussed above, performance of a single cell classifier on a multi-cell image can be improved if the assumption can be made that all cells in the field should show the same pattern. This can be done by assigning the most frequent class in the image to all cells [6]. While this assumption may be true in some cases, it is quite restrictive. The goal of the work in this paper is to improve the analysis of multi-cell images without the drastic assumption of homogeneity. We begin by considering a variation on this assumption, namely that each multi-cell image is composed of a small number of classes with roughly equal numbers of cells. In this case, one strategy is to decide upon the number of classes using a threshold on the observed frequencies of each class. We define *T*_*n *_= 1/(1 + *n*) + *β*, where *n *is the number of classes and *β *is an adjustable parameter that ranges from -0.5 to 0.5. To find the number of classes, we find the smallest *n *for which the frequencies of exactly *n *classes are greater than *T*_*n *_and record which classes those are. This definition is based on the assumption that the true classes are present in roughly equal proportion, and hence that the percentage of each should be greater than the expected frequency of a class if one more true class was present (plus a tolerance controlled by *β*). We consider an example to illustrate the approach. Using *β *= 0.1 results in *T*_1 _= 0.6 and *T*_2 _= 0.43. Given a field with three classes with frequencies (0.7,0.2,0.1), we would choose *n *= 1. However, if the frequencies were (0.45,0.5,0.05), *n *= 2 would be chosen. Once *n *is chosen, each cell in the trial field is assigned to the one of those classes that has the largest likelihood for that cell (as assigned by the single cell classifier). Note that this might not be the class with the highest likelihood if that class was not retained during the selection of the number of classes. If no *n *meets the criterion, we simply keep the classification results from the single cell classifier. Note that as *β *decreases to -0.5, we increasingly favor finding only one class, and as *β *approaches 0.5 we increasingly favor making no changes to the original class assignments.

### Evaluation scheme

To illustrate and test approaches to multi-cell classification, we need multi-cell images in which the class of each cell is known with certainty. Since it is nearly impossible to collect such images (without, for example, using micro-manipulation to spot cells on a slide), we have simulated them by combining images from a large library of single cell images (the 2D HeLa cell image collection described in the Methods). The library contains images of ten subcellular pattern classes, and to classify individual cells we have used a multi-class support vector machine classifier whose outputs were converted to probabilities for each class.

**Figure 2:**
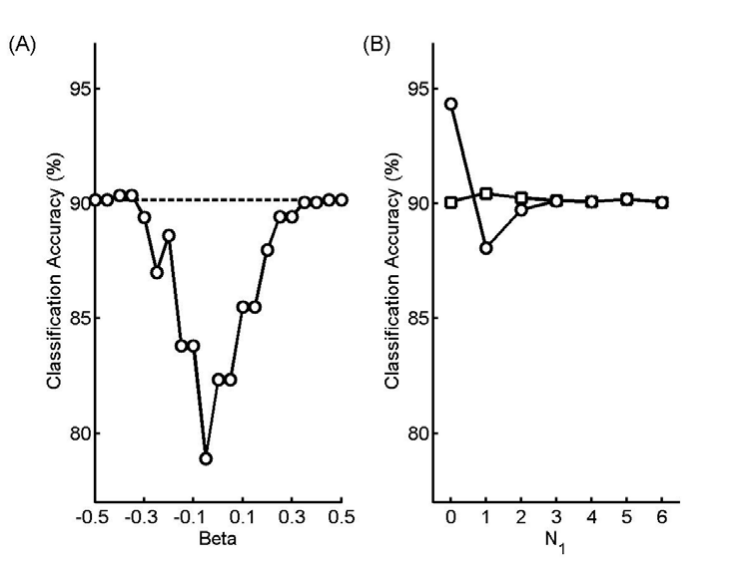
**Classification Accuracy for simulated fields of cells using the equal-sized class model**. Simulated fields consisting of N_1 _cells from one class and N_2 _cells from a different class were generated as described in the text, with N_1 _+ N_2 _= 12. A) The average accuracy across all N_1 _values are shown for the equal-sized class model (○) as a function of the model parameter *β*. The accuracy of the base single cell classifier is shown by the dashed line. The best accuracy (90.4%) of the equal-sized class model is obtained when *β *= -0.4. B) The improvement in average classification accuracy over the base single cell classifier is shown as a function of N_1_. Each point shows the average classification accuracy over 10 repeated trials for 12 cells for all possible pairs of classes for *β *= -0.4. The average accuracy of the equal-sized class model (○) and base classifier (□) are shown. The classification accuracy is better than that of the base classifier only when N_1 _= 0, the case when the set consists of just one class.

For the first tests, we created synthetic images consisting of 12 cells randomly drawn from only two classes such that the number of images in one class varied from 6 to 12. Average accuracies over 10 repeated trials were determined for the (base) single-cell classifier and for the equal-size class model described above. This process was repeated for all possible pairs of classes and for different mixtures of images from the two classes, and the average classification accuracy across all of these conditions was determined for various values of *β*. Figure 2a compares the overall classification accuracy across all mixtures between the base classifier and the equal-sized class model. The best average accuracy (90.4%) is obtained for *β *= -0.4. Figure 2b compares the classification accuracy for *β *= -0.4 between the base classifier and the equal-sized class model as a function of N_1_, the number of cells in one of the two classes. The classification accuracy is only better than that of the base classifier for the set consisting of only one class, but in all other cases the classification accuracies are either lower or equal. The results also indicate that cases of different mixtures need different optimal *β *s to achieve the best accuracy improvement (data not shown). For example, when N_1 _= 0, the accuracy can be improved up to 9.8% over the base classifier for *β *= -0.05, but the average accuracy across all mixtures is much worse (78.9%). The best accuracy improvements for cases with N_1 _= (1, 5, 6) are (1.1%, 1.9%, 2.7%) with *β *= (-0.15, -0.20, -0.20). However, for cases with N_1 _= (2, 3, 4), no matter how the *β *is tuned, the best possible average accuracy can only be the accuracy from the base classifier. This is expected since the assumption used to derive the method was that whatever classes are present are approximately equal in frequency. All these results suggest that the equal-size method should not be used when the mixture of classes is unknown.

### Construction of graphical models

We next consider what information may be available about the likely class of a cell given information about its neighbors in the field, and how we can construct a graphical model to use that information. Two limit cases can be considered. These limits are based on the relative magnitudes of the constants *t*_*plate *_and *t*_*g *_defined in the problem statement above.

#### Feature space model

The first possibility is that *t*_*plate *_is short relative to *t*_*g *_such that cells would not have time to undergo significant cell division prior to their being imaged. In this case, the proximity of cells does not provide any information about their likely similarity (i.e., whether they are derived from the same class). The only clues that we have about the number of classes present (and the number of cells in each) are the similarities between cells in the SLF feature space. In this case, we initially construct an undirected graph in which each cell is represented by a node and edges are created between each pair of nodes with length equal to the z-scored Euclidean distance between the feature vectors of the corresponding cells.

#### Physical space model

If, however, the amount of time that elapses between plating and imaging is significantly greater than the generation time (*t*_*plate *_≫ *t*_*g*_), each original cell is expected to have divided a number of times and we may consider it likely that the class of cells adjacent to one another is the same. The rate (*v*_*trans*_) at which daughter cells move away from each other relative to the rate at which they divide becomes the determining factor. Thus, if *v*_*trans *_is high, we may consider physical proximity to be of little predictive value and are forced to use the feature space model described above. If, on the other hand, *v*_*trans *_is low, we can construct an undirected graph using the Euclidean distance between the centers of cells in the field.

#### Pruning

Initially, the graphs for both model types are fully connected. Each edge suggests the two nodes it connects should influence each other's labels. Since we can assume that they should not influence each other if the distance between them is too large (and to improve computational efficiency), edges whose length is greater than a free parameter *d*_*cutoff *_are removed. Note that the units of *d*_*cutoff *_are different for the two types of models.

### Inference by prior updating

**Figure 3:**
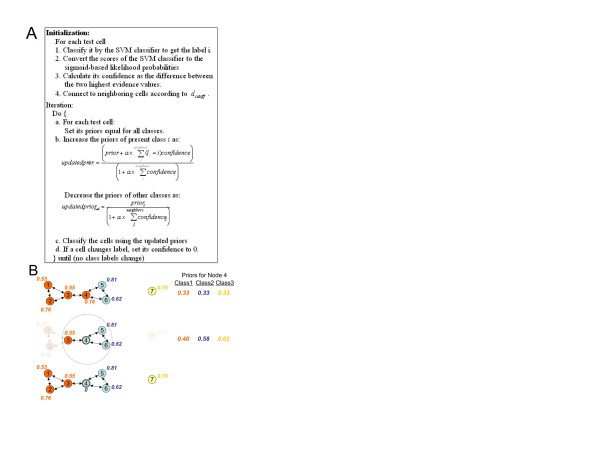
**The prior updating algorithm**. A) Pseudo-code for the algorithm is shown. A free parameter *α *in the updating equation is used to determine the degree of change of priors. When *α *is zero, the priors do not change and the graphical model results are the same as the results of the base classifier. The priors are pushed harder to the majority classes in the field as *α *increases. B) Illustration of the PU algorithm for a graphical network of seven cells and three classes.

Given a graphical model of either of these types, the task becomes inferring the class labels. This requires an algorithm to describe how the label at each node is influenced by information from each of its nodes. As described in the introduction, exact and even approximate inference methods can be extremely compute intensive for models with many connected nodes. Since our goal is to apply graphical models to fields with many cells, we need an efficient method for inferring the most likely class for each cell given the results of the single cell classifier for it and its neighbors. We therefore developed a new method, which we term prior updating (PU), that we believed could give improved classification accuracy in realistic compute times. The principle behind the method is simple: we allow each cell to have its own set of prior probabilities for all possible classes and adjust them to reflect the likely classes of its neighbors. We start by setting all prior probabilities equal, and then determine the posterior probability of each class for each cell using the output of the SVM classifier and Bayes rule. We then iteratively adjust the prior probabilities of all classes for each cell based on the labels of its neighbors and recalculate the posterior probabilities (Figure 3). A free parameter *α *controls the extent to which the prior probabilities are adjusted at each iteration (for *α *= 0, no adjustment is made). The method terminates when no class labels change during an iteration. Each cell is allowed to change its label at most once, and its confidence is set to zero after the label changes. We designed this strategy because cells whose labels are easily changed are expected to have high uncertainty, and should not influence other cells after their labels change. This strategy also guarantees that the iteration will converge in constant time. Similar results are obtained if priors for each node are initialized outside the loop and if labels are allowed to change more than once (data not shown).

#### Feature space model

**Figure 4:**
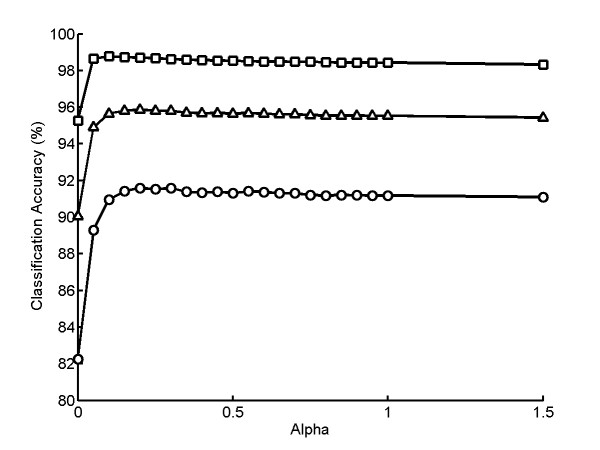
**Improvement of classification accuracy using feature space graphical model**. Each point shows the average classification accuracy over 10 repeated trials for 12 cells for fields of six cells each from two classes. The average accuracy for pairs of similar classes (○), dissimilar classes by (□), and all classes (△) are shown. The best accuracies are obtained with *α *= 0.15 and *d*_*cutoff *_= 8. The accuracy of similar classes is improved by 9% (from 82.2% to 91.3%), while the accuracy of dissimilar classes is also improved 3% (from 95.3% to 98.5%). The overall accuracy is improved by the prior updating method by over 5%(from 90.1% to 95.7%).

To evaluate the accuracy of the graphical models and the prior updating method, we used synthetic multi-cell images as described above. We first consider the feature space model, which is directly comparable to the equal-size scheme since neither considers physical position of a cell. We calculated classification accuracy for various values of the two free model parameters: *α *and *d*_*cutoff*_. Figure 4 shows results for fields of 6 cells each for two classes for the best *d*_*cutoff *_for each of various values of *α*. The best results were obtained with *α *= 0.15 and *d*_*cutoff *_= 8. We evaluated three metrics: overall accuracy (average of all 10 classes), average accuracy for similar classes (the endosomal and lysosomal proteins and the two Golgi proteins), and accuracy for dissimilar classes (the remaining classes). Compared with the results for the base classifier (without inference), the accuracy of similar classes is much improved (by 9 percentage points, from 82.2% to 91.3%), and the accuracy of dissimilar classes is also improved (by 3 percentage points, from 95.3% to 98.5%). The overall accuracy is improved by over 5 percentage points (from 90.1% to 95.7%). The overall accuracy of 95.7% obtained with an SVM classifier combined with PU is higher than the best previous accuracy for the 2D HeLa collection of 92.3%, which was obtained using a much more complicated majority-voting classifier [10].

When *α *is zero, the priors are not updated so that cells do not influence each other. As *α *increases, the priors of classes that are present in the field are increased while others are decreased. As seen in Figure 4, classification accuracy also increases as *α *increases but roughly plateaus at *α *near 0.2. The results suggest that a large *α *usually gives good improvement in classification accuracy; however, the best *α *has to be found by applying cross-validation methods.

The *d*_*cutoff *_parameter is designed to determine the neighbors of a cell. If *d*_*cutoff *_is very small, the cell does not have any neighbors to influence and be influenced by. As *d*_*cutoff *_gets larger, the cells start to be influenced by other similar cells, and so the classification accuracy can be improved. If *d*_*cutoff *_is set to infinity, all the cells are connected to each other in the graph and so contribute to the updates of each other's priors. In this case, some dissimilar cells will affect each other's priors and the classification accuracy could be worse than when the best *d*_*cutoff *_is used. The best *d*_*cutoff *_can be found by applying cross-validation methods.

**Figure 5:**
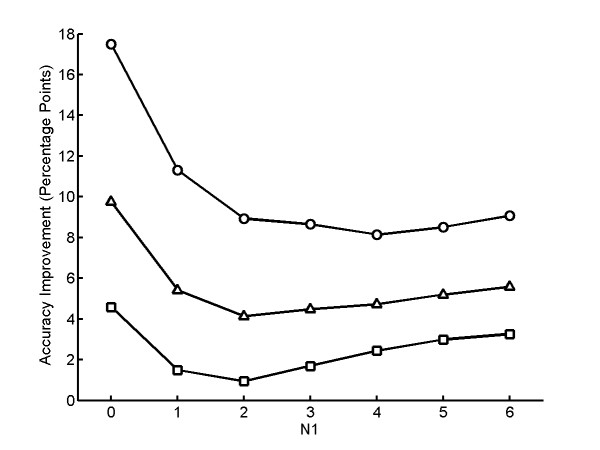
**Improvement in classification accuracy for simulated fields of cells using a feature space graphical model**. Simulated fields consisting of N_1 _cells from one class and N_2 _cells from a different class were generated as described in the text. A class label was assigned to each cell in the simulation using the feature space graphical model described in the text. The improvement in average classification accuracy over the base single cell classifier is shown as a function of N_1_, where N_1_+N_2 _= 12. Each point shows the average classification accuracy over 10 repeated trials for 12 cells for all possible pairs of classes. The average accuracy for pairs of similar classes (○), dissimilar classes by (□), and all classes (△) are shown. Results except for N_1 _= 0 are for a *d*_*cutoff *_value of 8, the best value of those tested.

Encouraged by these results, we evaluated trial fields with two classes of varying numbers of cells in the feature space field (Figure 5). For the N_1 _= 0 case, where there is only one class of cells present in the field, the best *d*_*cutoff *_and *α *are both infinite, so that all the cells can be classified into one class just as the equal-sized class scheme does. The best *d*_*cutoff *_is 8 for all other cases. This implies that the z-score distances among similar cells of 2D HeLa images in the SLF16 feature space are on average less then 8, no matter how many cells the classes are composed of. The best *α *ranged from 0.2 to 0.5 for different cases (data not shown). The results in Figure 5 were obtained with *α *set to 0.5, and this value was used for all subsequent experiments. As the sizes of the two classes become more asymmetric (from N_1 _= 6 to N_1 _= 2 case), the accuracy improvement of similar classes still remains in the range of 8 to 9 percentage points, while the accuracy improvement of dissimilar classes decreases from 1 to 3 percentage points. This is because smaller numbers of "minority" classes affect the estimated priors to a lesser degree, and a small change in priors is more likely to affect the labels of similar classes than of dissimilar ones. For the N_1 _= 0 and N_1 _= 1 case, the accuracy of similar classes are higher than for the other cases, which confirms that it is easier to determine which of similar classes a cell is more likely to be when the cells are more homogeneous in the field.

#### Physical space model

**Figure 6:**
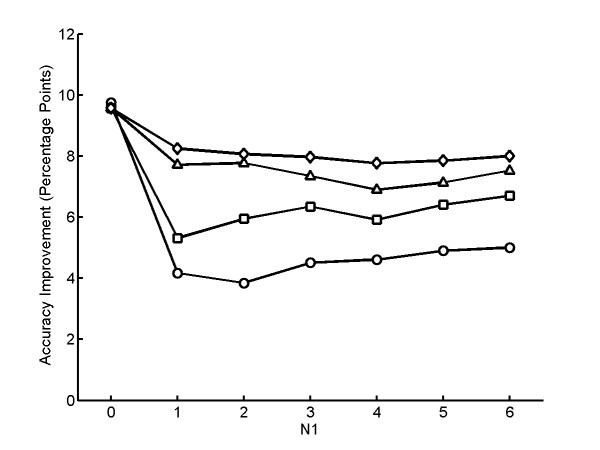
**Improvement in classification accuracy for simulated fields of cells using a physical space graphical model**. Simulated fields containing clones of cells consisting of N_1 _cells from one class and N_2 _cells from a different class were created as described in the text for various values of D, the distance between the initial cells of each class. A class label was assigned to each cell in the simulation using the physical space graphical model described in the text. The improvement in average classification accuracy over the base single cell classifier is shown as a function of N_1_, where N_1_+N_2 _= 12. Each point shows the average classification accuracy over 10 repeated trials for 12 cells for all possible pairs of classes for fields generated with D = 0 (○), D = 6 (□), D = 12 (△), and D = 400 (◇). Results except for N_1 _= 0 are for a *d*_*cutoff *_value of 6, the best value of those tested. Note that, as expected, the accuracy improves with increasing D.

We also evaluated a model in which the physical positions of cells in the field are used to influence classification. Synthetic fields of cells were created by simulating the processes of cell division and movement for clones derived from two cells of different classes initially at a distance *D *from each other. Figure 6 shows results for applying the graphical models on fields generated with various values of *D*. When *D *= 0, the two clones overlap in space but in most cases, the accuracies for similar and dissimilar classes are still improved over the base classifier. This is expected, since this case is very similar to the feature space model evaluated above. The classification accuracy improves as the separation of the two clones increases (*D *> 0), also as may be expected. The results demonstrate the important conclusion that our graphical models can result in significant improvement in classification accuracy for the task of classifying a mixed population of cells under a variety of test conditions.

### Multiple classes test

Given that our method performed well for multiple cells from two different classes, we next examined the cases where more than two classes of cells were present. We therefore performed experiments using the feature space model on one to five classes with each class having six cells. The results are shown in Table 1. As the number of classes increases, the overall accuracy decreases from around 9 percentage points *above *to around 2 percentage points *below *that of the base classifier. Since it is more likely that there are cells from both of two similar classes in the field as the number of classes increases, this is expected. The observation that the transition from improvement to degradation occurs after 4 out of 10 classes are present loosely suggest that the maximum number of classes that can be simultaneously present in a field and still see improvement from a graphical model is around 40% of the number of possible classes.

**Table 1:** Results for multiple classes.

	Classification Accuracy (%)		
No. of classes	Similar Classes	Dissimilar Classes	All Classes

1	96.7	99.8	98.6
2	91.3	98.5	95.6
3	86.4	97.3	92.9
4	82.0	96.0	90.4
5	78.2	94.8	88.1
Base Classifier	82.2	95.3	90.1

The accuracy of classification using feature space graphical models were evaluated for all possible mixtures composed of various numbers of classes drawn from ten classes. Results shown are averages over 10 trials.

### Effect of training set size

We also examined the effect of training set size on the prior updating scheme as a way of examining the improvement possible for a less accurate base classifier. Various training set sizes were used to train base classifier SVMs and then these were applied to fields of two classes of equal sizes. The results in Table 2 show that the base SVM classifier decreases its accuracy with fewer training data (as expected), but that the prior updating scheme can still improve its accuracy by between 5 and 8 percentage points. The smaller the amount of the training data, the more the prior updating method can improve and compensate the accuracy. It is especially impressive that the combination of the prior updating scheme and the SVM classifier with only 10 training data per class can do a similar job to the SVM classifier alone with 50 training data per class. The results also indicate that at least for this subcellular pattern classification task, the SVM classifier joined with the prior updating scheme does not need a lot of training data in order to attain a fair classification performance.

**Table 2:** Results for different training set sizes.

	Classification Accuracy (%)					
No. of training data	Similar Classes		Dissimilar Classes		All Classes	
	
	No updating	With updating	No updating	With updating	No updating	With updating

50	82.2	91.3	95.3	98.5	90.1	95.6
40	80.8	90.2	94.9	98.3	89.2	95.1
30	78.9	88.9	94.2	98.4	88.1	94.6
20	76.3	87.5	93.2	98.0	86.4	93.8
10	71.2	80.8	90.6	96.6	82.9	90.3

Base SVM classifiers were trained using various numbers of cells for each of ten classes. Graphical models using those base classifiers were then tested on fields containing six cells each from two classes and results were averaged over 10 trials each for all possible pairs.

## Discussion

Our work has particular implications for classification of patterns in images obtained by high-throughput microscopy. Since high-throughput systems typically use low magnification, the number of cells per field is often high and the accuracy of single-cell classifiers is usually not perfect. By applying this method on multi-cell images made of real single cells and synthesized locations, we are able to verify that our scheme can be used for such systems to achieve significantly better performance.

Since we have proposed a new approximate inference algorithm, it is important to identify when this method works better than other approximate inference methods. This method is very fast compared to previously described graphical model algorithms: its runtime is linearly proportional to the number of cells in each trial field and to the number of classes it needs to choose from. Whether this method has better classification performance under different circumstances will be examined in future work. We anticipate that the method can be made more general so that it can be used for other applications, both for biomedical applications like classification of cell types in tissue images and for other applications like Internet link analysis.

## Conclusion

This paper addresses a supervised learning problem in the domain of protein subcellular location determination. We have proposed a novel graphical representation where multiple cells in a field influence each other. Assuming that these cells are only composed of a small number of classes, the classification accuracies are improved by manipulating the prior distributions of classes. The improvement is largest for groups of classes which would be difficult for the base classifier to distinguish from one another.

We have also shown the robustness of our prior updating scheme. The accuracies for different classes were always improved under different assumptions about the distribution of cells in the field, different sizes of the two classes of cells present in the field, different numbers of classes, and different training set sizes.

The results are very encouraging since the prior updating method improves the overall accuracy from the base classifier by around 5 percentage points and the accuracy of similar classes by around 9 percentage points. The combination of the prior updating method and the base single cell classifier outperforms the majority voting classifier that with an accuracy of 92.3% had the best prior reported performance on this dataset [10].

## Methods

### 2D HeLa cell image collection

**Figure 7:**
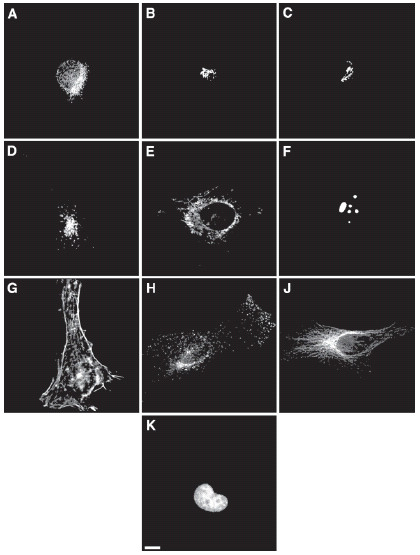
**Typical images from the 2-D HeLa cell image collection used in this study**. Images are shown for cells labelled with antibodies against an ER protein (A), the Golgi protein giantin (B), the Golgi protein GPP130 (C), the lysosomal protein LAMP2 (D), a mitochondrial protein (E), the nucleolar protein nucleolin (F), transferring receptor (H), and the cytoskeletal protein tubulin (J). Images are also shown for filamentous actin labelled with rhodamine-phalloidin (G) and DNA labelled with DAPI (K). Scale bar = 10 μ*m*. From [6].

The 2D HeLa cell image collection was created by introducing antibodies and molecular probes against proteins in major subcellular organelles [6]. This data set contains 862 single-cell images consisting of ten classes, each of which contains from 73 to 98 images. Figure 7 shows typical images from each class. Every image has a resolution of 382 × 512 pixels and each pixel represents 0.23 × 0.23 μm in the sample plane. In parallel to each protein image, an image of the DNA distribution was obtained using a DNA-specific fluorescent probe. These parallel images provide a common reference framework for describing the distribution of each protein.

### Subcellular Location Features (SLF)

We have developed several sets of informative features to describe protein subcellular patterns. These features, termed Subcellular Location Features (SLFs), are of several types, including Zernike moment features, Haralick texture features, morphological features and wavelet features. The details for different versions of SLFs are reviewed in [10]. The best classification results obtained to date for the 2D HeLa dataset were with feature set SLF16 [10], and we have therefore used the SLF16 feature set in this work. Each cell in the dataset is thus represented by a feature vector *x *of length *d *= 47.

### Bayesian decision theory

Bayesian decision theory is a fundamental statistical approach to pattern classification problems [24]. The Bayes formula can be expressed as:

p(wj|x)=p(x|wj)p(wj)p(x)
 MathType@MTEF@5@5@+=feaafiart1ev1aaatCvAUfKttLearuWrP9MDH5MBPbIqV92AaeXatLxBI9gBaebbnrfifHhDYfgasaacH8akY=wiFfYdH8Gipec8Eeeu0xXdbba9frFj0=OqFfea0dXdd9vqai=hGuQ8kuc9pgc9s8qqaq=dirpe0xb9q8qiLsFr0=vr0=vr0dc8meaabaqaciaacaGaaeqabaqabeGadaaakeaacqWGWbaCcqGGOaakcqWG3bWDdaWgaaWcbaGaemOAaOgabeaakiabcYha8jabdIha4jabcMcaPiabg2da9maalaaabaGaemiCaaNaeiikaGIaemiEaGNaeiiFaWNaem4DaC3aaSbaaSqaaiabdQgaQbqabaGccqGGPaqkcqWGWbaCcqGGOaakcqWG3bWDdaWgaaWcbaGaemOAaOgabeaakiabcMcaPaqaaiabdchaWjabcIcaOiabdIha4jabcMcaPaaaaaa@4AB7@

where *w*_*j *_is the class with index *j*, *p*(*w*_*j*_), termed the prior probability, is the probability of class *j *being observed in the absence of any other information, *p*(*x | w*_*j*_), termed the likelihood probability, is the probably density function for an observed feature vector *x *given that the class is *w*_*j*_, *p*(*w*_*j *_| *x*), termed the posterior probability, is the probability of the class being *w*_*j *_given that *x *has been observed, and *p*(*x*), termed the evidence, is just a normalization to guarantee that the posterior probabilities sum to one. For *n *classes, the evidence can be formulated as

p(x)=∑j=1np(x|wj)p(wj).
 MathType@MTEF@5@5@+=feaafiart1ev1aaatCvAUfKttLearuWrP9MDH5MBPbIqV92AaeXatLxBI9gBaebbnrfifHhDYfgasaacH8akY=wiFfYdH8Gipec8Eeeu0xXdbba9frFj0=OqFfea0dXdd9vqai=hGuQ8kuc9pgc9s8qqaq=dirpe0xb9q8qiLsFr0=vr0=vr0dc8meaabaqaciaacaGaaeqabaqabeGadaaakeaacqWGWbaCcqGGOaakcqWG4baEcqGGPaqkcqGH9aqpdaaeWbqaaiabdchaWjabcIcaOiabdIha4jabcYha8jabdEha3naaBaaaleaacqWGQbGAaeqaaOGaeiykaKIaemiCaaNaeiikaGIaem4DaC3aaSbaaSqaaiabdQgaQbqabaGccqGGPaqkaSqaaiabdQgaQjabg2da9iabigdaXaqaaiabd6gaUbqdcqGHris5aOGaeiOla4caaa@4971@

A probabilistic classifier assigns an observation *x *to class *i *if

*p*(*w*_*i *_| *x*) > *p*(*w*_*j *_| *x*)     ∀*j *≠ *i*

That is, the classifier assigns *x *to the class with the maximum posterior probability.

In our previous work, each cell was classified independently. Since the priors were not known in advance, they were assumed to be equal. In this case, the classification with the "Maximum a Posteriori Probability" (MAP) is equivalent to the "Maximum Likelihood" (ML).

### Classifier – Support Vector Machine

Support Vector Machines (SVM) were originally designed for binary classification by finding a maximum margin hyperplane between two classes [25]. They can be extended to solve multi-class classification problems by combining several binary classifiers. There are several commonly used methods, such as one-against-all, one-against-one, and directed acyclic graph. Here we adapt the one-against-all method [26,27], which constructs *n *SVM classifiers where *n *is the number of classes. The *i*_th _SVM is trained using all of the examples in the *i*_th _class with positive labels and all others with negative labels. The test example is fed into these *n *SVMs and the one with the highest output score is selected as the final class. Each SVM used an exponential radial basis function kernel with *C *= 20 and *σ *= 7, where *C *mediates the trade-off between maximizing the margin and minimizing the training error, and *σ *is the parameter in the expression:

K(x,y)=e−(x−y)22σ2
 MathType@MTEF@5@5@+=feaafiart1ev1aaatCvAUfKttLearuWrP9MDH5MBPbIqV92AaeXatLxBI9gBaebbnrfifHhDYfgasaacH8akY=wiFfYdH8Gipec8Eeeu0xXdbba9frFj0=OqFfea0dXdd9vqai=hGuQ8kuc9pgc9s8qqaq=dirpe0xb9q8qiLsFr0=vr0=vr0dc8meaabaqaciaacaGaaeqabaqabeGadaaakeaacqWGlbWscqGGOaakcqWG4baEcqGGSaalcqWG5bqEcqGGPaqkcqGH9aqpcqWGLbqzdaahaaWcbeqaamaalaaabaGaeyOeI0IaeiikaGIaemiEaGNaeyOeI0IaemyEaKNaeiykaKYaaWbaaWqabeaacqaIYaGmaaaaleaacqaIYaGmiiGacqWFdpWCdaahaaadbeqaaiabikdaYaaaaaaaaaaa@416E@

The kernel function K is a distance function for two feature vectors *x *and *y*. The multi-class SVM produces uncalibrated scores that are expected to be positively correlated with the confidence of the assignment but which are not directly comparable between classes. Thus, we use a sigmoid function to calibrate the output scores of the SVM. The parameters of the function can be found by minimizing the negative log likelihood of the training data [28]. The resulting probabilities are then comparable between different classes. We associate with each node an evidence vector consisting of the probabilities for each class and a label corresponding to the class with largest evidence. The confidence of this label is defined as the difference between the two highest class probabilities.

### Creation of synthetic multi-cell images

To synthesize multi-cell images, we used the 2D HeLa image set composed of 10 classes of major subcellular location patterns (described above). To meet the assumptions that cells are only composed of a small number of classes, we constructed trial fields consisting of cells drawn from all possible pairs of the 10 classes in the 2D HeLa dataset. For each trial, N_1 _and N_2 _cells were randomly picked from two different classes with total number of 12 cells. Separate trials were conducted for N_1 _from 0 to 6.

For cross-validation, we split the data into five folds: one fold for the testing pool and the other four folds for the training pool. In the training pool, 50 images from each class were randomly chosen and for each trial, N_1 _and N_2 _cells were randomly picked from all possible pairs of classes out of the testing pool. Each of the five folds was in turn used for testing and the remaining four for training a multi-class SVM classifier. The classification accuracies were averaged for each pair of classes over all five folds. Some of the images are used neither for training nor for testing in any one fold, but the testing images may be used more than once overall due to lack of data. Because of this reuse, this evaluation method is similar to the usual five-fold cross validation procedure but not the same. In expectation it will report the correct accuracy for the classifier, but the variance of its reported accuracy is difficult to compute. To reduce this variance as much as possible we average 10 trials by randomly assigning images in the testing and training pools.

**Figure 8:**
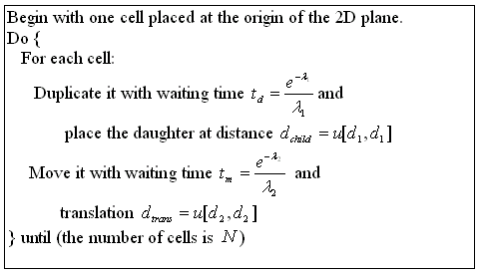
**Algorithm for simulating cell fields**. The algorithm simulates the formation of a clone of *N *cells from a single cell and incorporates cell growth and movement. *u[d,d] *represents a two dimensional uniform distribution from -*d *to *d *(e.g., a cell can move to anywhere within the square with length of the side equals to *2d*). *d*_1 _and *d*_2 _describe how much cells spread apart after cell division. *t*_*d *_corresponds to the average generation time of *t*_*g*_, and *t*_*m *_indicates the average time a cell moves. If the *d*_1 _and *d*_2 _are the same, large *t*_*d *_and small *t*_*m *_will result a more compact colony, while small *t*_*d *_and large *t*_*m *_will result a sparser colony.

**Figure 9:**
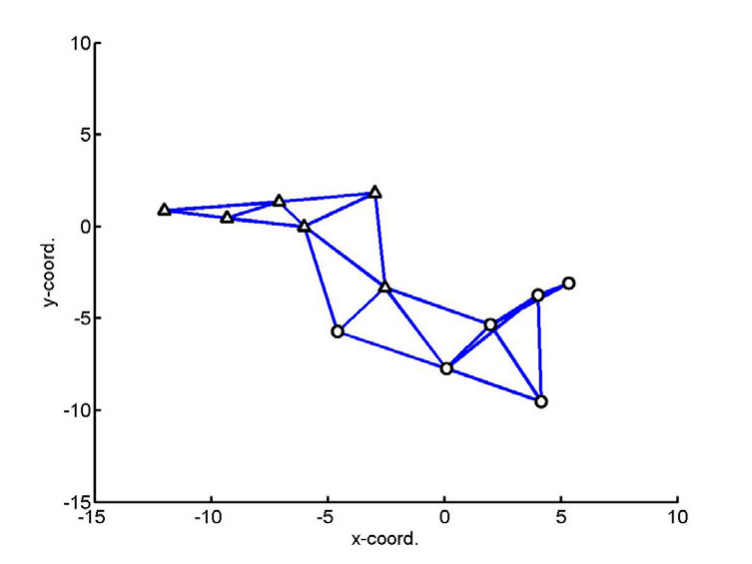
**Simulation of cell positions for two classes**. Two simulated clones from different classes were generated with a separation parameter *D *defining the distance between the initial cell positions. An example of the distribution for two simulated clones of six cells each is shown for D = 12. Edges connect cells that are less than 6 units apart. Note that some of these edges connect cells from different classes.

Since the 2D HeLa images were originally collected for single cells without recording their position on the slide, we simulated the positions of the cells according to a simple model of cell growth and movement. The pseudo-code of the simulation is shown in Figure 8. Once the positions were simulated, a randomly-chosen cell from a specified class of the HeLa images was assigned to each position. To simulate the presence of more than one class on a slide, two (or more) simulated clones from different classes were generated with a separation parameter *D *representing the distance between their origins. An example for two clones of six cells each is shown in Figure 9, with edges drawn between cells that are less than 6 units apart (i.e., *d*_*cutoff *_= 6).

### Code availability

The data and source code used for the work described in this paper is available from .

## Authors' contributions

SCC participated in the design of, and carried out, all experiments, and drafted the manuscript. RFM conceived of the approach, participated in its design and coordination, and helped extensively with writing the manuscript. Both authors read and approved the final manuscript.
